# Animal Control and Field Services Officers’ Perspectives on Community Engagement: A Qualitative Phenomenology Study

**DOI:** 10.3390/ani13010068

**Published:** 2022-12-24

**Authors:** Liana R. Moss, Sloane M. Hawes, Katherine Connolly, Morgan Bergstrom, Kaleigh O’Reilly, Kevin N. Morris

**Affiliations:** Institute for Human-Animal Connection, Graduate School of Social Work, University of Denver, Denver, CO 80208, USA

**Keywords:** community engagement, animal control, field services, animal protection, animal cruelty, animal neglect

## Abstract

**Simple Summary:**

There is a critical gap in the literature regarding how community engagement is employed to address animal cruelty and neglect. This study sought to understand community engagement from the perspective of animal control and field services officers. The results revealed that definitions of community engagement varied greatly among the officers interviewed. Participants shared barriers and best practices in their current community engagement efforts as well as their wishes for future directions of the field. Subsequent studies should seek to establish a consistent definition of community engagement in animal control and field services that can then be tailored to specific communities.

**Abstract:**

Very little is known about the prevalence, scope, and methods of community engagement employed by animal control and field services officers to address the issue of animal cruelty and neglect. This study used a phenomenological approach to understand how officers defined community engagement. The researchers conducted semi-structured interviews with twenty-nine animal control and field services officers. The definitions of community engagement varied greatly across this sample of U.S. officers. However, most officers agreed that strategies such as relationship-building, providing assistance or information, and allowing time for compliance were among the most effective community engagement strategies. In addition, several barriers to incorporating community engagement strategies in the work of animal control professionals were identified. Future research and policymaking should seek to establish a consistent definition of community engagement in animal control and field services that can then be optimized for specific communities through rigorous evaluation.

## 1. Introduction

Community engagement has been used across a broad spectrum of social issues to develop interventions by identifying the needs and priorities of focus communities [1,2]. By providing community members the opportunity to collaboratively identify issues and offer critical perspectives in shaping the policies and practices that directly impact them, effective community engagement can identify individual and collective strengths, challenges, assets, and lived experiences on the issues that affect community members [1,2]. Engaging and leveraging existing community networks is also essential to putting identified policies into successful practice. Overall, community engagement allows agencies to establish relationships with key stakeholders in their community so goals can be achieved, and issues effectively addressed [2,3]. Because animal cruelty and neglect can be affected by community-specific definitions, practices, and barriers to accessing pet-supportive services, its resolution could benefit from effective community engagement approaches. 

Animal control and field services officers (also often referred to as animal protection and animal services officers) are on the front lines of many animal protection issues, putting them in a unique position to engage with the communities they serve. Animal protection encompasses a “broad spectrum of political, legal, economic, and investigative strategies” [4]. For the purpose of this study, the authors utilize the terms “animal control” and “field services” to describe this concept because they are the most commonly used terms in the United States to describe the professionals who are tasked with animal protection efforts, including, but may not be limited to: enforcement of animal-related laws and ordinances, management of dangerous or vicious animals, reuniting lost people and their pets, addressing animal specific public health concerns (rabies, animal waste, etc.), and investigation of animal neglect, cruelty, hoarding, and intentional acts of abuse [5]. In the United States, animal protection activities are carried out by a variety of agency types including municipal agencies, nonprofit agencies with a government contract, and nonprofit agencies without a government contract [4].

To empower communities to prevent and respond to animal cruelty and neglect, animal control and field services organizations must employ effective and culturally responsive community engagement strategies [6]. These strategies include efforts to address systemic and sociocultural barriers to accessing pet support services (e.g., veterinary care, behavior care, and basic supplies) [7,8]. While the animal welfare field has made progress in conducting research on approaches to address access to pet support services over the last five years, there is little to no evidence informing best practices for community engagement on animal cruelty and neglect issues [9,10,11,12,13,14]. Several recent studies have discussed how effective community engagement strategies are critical to addressing the underlying causes of animal cruelty and neglect, yet no research has specifically evaluated which community engagement strategies are most effective for this particular issue [8,9,10,11,12,13,14,15]. Very little is known about the prevalence, scope, and methods of community engagement employed to address the issue of animal cruelty and neglect. Therefore, in partnership with the National Animal Care & Control Association (NACA), the present qualitative study sought to understand how animal control and field services officers defined, understood, and practiced community engagement in their work. NACA is a national membership organization for animal control and field services professionals that was formed in the United States in 1978 and is “committed to setting the standard of professionalism in animal welfare and public safety through training, networking, and advocacy” [16]. Leveraging these officers’ perspectives in developing future policies and programs to promote animal wellbeing is essential to understanding and addressing systemic inequities and creating sustained change.

## 2. Materials and Methods

### 2.1. Design 

This study used Gadamer’s hermeneutical phenomenology to understand how animal control and field services officers defined community engagement in their work. Phenomenology allows researchers to develop a common meaning of a phenomenon based on a group of individuals’ lived experiences [17]. Gadamer’s hermeneutical phenomenology is typically used “to reveal the unseen world of lived experiences” on a topic, particularly when there is limited research on the subject [18]. There are three primary philosophical foundations of Gadamer’s hermeneutical phenomenology: acknowledging researcher pre-understandings, engaging in a “fusion of horizons,” and establishing the “hermeneutic circle.” [19]. Prior to engaging in data collection for this study, the research team took time to share and reflect on their own lived experiences around community engagement (“pre-understandings”) and were instructed to continuously reflect on how and where their meanings aligned with and diverged from the perspectives shared by the officers in the study. Through this method, the research team’s multidisciplinary background (e.g., animal protection; community organizing; community outreach) with knowledge and lived experience of how community engagement has been implemented in a variety of other contexts could also be leveraged as a way to understand and interpret the experiences of the research participants more deeply [19].

Recognizing that the topic of engaging with community members on issues of harm to animals was likely to be sensitive and challenging to speak about, which could ultimately impact the comprehensiveness of the findings, the research team also took time prior to engaging in data collection to evaluate the research procedures with attention to trauma-informed care principles and the goal of minimizing harm for both the participants and the researchers that could result from participation in the study [20]. This involved acknowledging the privilege and power inherent to the researcher-participant dynamic and evaluating the interview questions for their ability to acknowledge and affirm the lived experiences of the officers. When administering the consent, the researchers took time to build rapport with the participants and emphasized their rights to decline to answer any question or withdraw from the study at any time. Teach-back consent was used to verify each participant’s comprehension of this consent information. 

### 2.2. Recruitment 

Recruitment for the study was carried out under a University of Denver Institutional Review Board-approved protocol (Protocol #1837024). To participate, interested participants needed to be at least 18 years old; be members of NACA; consent to being audio-recorded; and be currently employed as an animal control or field services officer. All NACA members (*n* = 1858) received an email from the research team with information regarding the study and a link to opt-in to participate. The opt-in form included the electronic consent form and basic demographic questions (race/ethnicity, city, state, gender identity, languages proficient in reading, writing, and/or speaking, years worked in animal control and field services, and type of organization). The electronic consent form included questions to verify participant comprehension of the study procedures. Participants were also provided contact information for the principal investigator so the participant could ask questions before consenting to participate. Participant consent forms were electronically signed and stored in a secure data management system hosted at the University of Denver (REDCap) [21]. The demographic information provided by the participants in the opt-in form was then used by the research team to assess the officers’ eligibility to participate in the study and then to select a diverse sample of NACA members for the interviews. For the purposes of this study, a “diverse” sample was defined as including as much diversity in the following identity groups as possible: gender identity, race, ethnicity, language proficiency (e.g., English, Spanish), geographic location, number of years working in the field, and organization type (e.g., municipal, non-profit with a government contract, non-profit without a government contract). Sixty-three NACA members completed the opt-in form. In order for participants to be included in the study, they had to be 18 years and older and consent for the study interview to be audio-recorded. Participants also had to be National Animal Care & Control Association members and currently work in animal control and field services. Thirty of the sixty-three respondents were selected to participate in the study based on these criteria. One of the selected respondents was unable to be reached by the research team after multiple attempts to contact them and, therefore, did not participate in the study.

### 2.3. Data Collection 

All consented study participants completed a one-hour, semi-structured interview between March 2022–May 2022 with a research team member by video conference (Zoom Video Communications, Inc.). The questions for the interview were drafted to facilitate the hermeneutic process of allowing each participant to reflect openly and deeply about their experiences with community engagement [19]. First, the researchers asked participants how they integrate community engagement strategies into their work. The following questions focused on suggestions for things to add, remove, or improve upon to better support their community engagement efforts (Appendix A). Participants were mailed a $50 Visa gift card after completing the interview as compensation for their time. Interviews were audio-recorded, then de-identified and stored in a password-protected database. A second-party vendor (GoTranscript, LTD) transcribed the de-identified audio recordings. To ensure accuracy, a research team member reviewed the transcript while listening to the recording and made corrections if there were any discrepancies between the transcript and the recording. These verified transcripts were used for analysis.

### 2.4. Data Analysis 

To achieve a rich and historically relevant account of the topic of study, Gadamer’s hermeneutical phenomenology requires that the researchers “be and stay open” during the data analysis process to an iterative process of developing a deeper understanding of the phenomena, including evaluating how they as researchers have come to experience the phenomena (“fusion of horizons”) [19,22,23,24]. This process of reciprocal reflection and interpretation assists researchers with not only “acquiring the commonalities between each story told, but also the contradictions that may arise,” (“hermeneutical circle”) [19]. There are no formal recommendations for how to structure analysis using Gadamer’s hermeneutical phenomenology, so the researchers consulted with other researchers who have applied the method to inform their analysis approach. The transcripts were systematically, deductively coded for themes. Memoing and group discussions throughout the coding process were guided by the philosophical foundations of Gadamer’s hermeneutical phenomenology [17,19]. First, the researchers read each transcript to glean the overall meaning of each individual participant’s experience (“immersion”). Second, the researchers generated interpretive summaries of the major topics of significance that arose in each individual interview [18]. Third, the researchers met as a group to discuss common themes across the interviews (“abstraction”). During this process, the themes were challenged, refined, and revised based on the other participants’ understandings and the researcher’s preunderstandings. Fourth, the themes were then entered as codes into a qualitative coding software (ATLAS.ti, Version 3.19.0; ATLAS.ti, GmbH, Berlin, Germany) to more efficiently facilitate the process of aggregating and grouping the themes according to common meanings. Fifth, a group of four researchers then applied the codes to each of the interview transcripts to allow the researchers to further investigate how each code differed based on the individual participants’ experiences. Lastly, the coded data were reviewed, and the final set of themes was organized and refined to illuminate the shared meaning of community engagement across the study participants (“aggregation” and “illumination”).

### 2.5. Validity and Trustworthiness 

Throughout the analysis, research personnel met for peer debrief sessions in order to challenge assumptions and biases and facilitate the formation of the “hermeneutic circle” by critically assessing interpretations of the data [18]. Findings were reported below using as many direct quotes as possible to allow the reader an opportunity to participate in validating the data [19] Finally, while not required for phenomenology, a summary of the themes was provided to research participants to review to ensure that the results aligned with their lived experiences and to gather input on potential implications of the findings [23,25]. Participants had the option to review the findings by phone (verbally) with a research team member or by email (written). All participants who reviewed the findings requested to review the findings by email. The email sent to participants included a secure link to a survey where they could anonymously review a summary of the themes and share if there was anything included in the description of the findings that they would change or add to better reflect their lived experience. Participants were also asked three open-ended questions on the implications of the findings: (1) Did the results from this study surprise you? Why or why not? (2) Do you think that these findings will impact your work? If yes, in what ways will they impact your work? (3) Do you think these results will have an impact on the field of animal welfare? If yes, in what ways? Three participants (10%) responded with their feedback on the summary of the findings and with responses to the open-ended questions on the implications of these findings, and their input was integrated into the results reported here. Because member-checking is not essential to phenomenology, the researchers did not have major concerns regarding the low response rate from participants at this stage of the study [19].

## 3. Results

### 3.1. Demographics 

Of the 29 participants, 16 (55%) self-identified as female, 12 (41%) as male, and 1 (3%) as transgender. In addition, 2 participants (7%) self-identified as Asian, 1 (3%) as Black, 2 (7%) as Hispanic/Latino/a/e, 23 (79%) as white, and 1 (3%) as two or more races. The participants were from 21 different U.S. states. Twenty-two (76%) participants worked at a municipal agency, 6 (21%) worked at a non-profit agency with a government contract, and 1 (3%) worked at another type of agency. The average number of years the participants had been working in the animal control and field services field was 13 years with a median of 12 years (minimum 1 year, maximum 40 years). A detailed summary of participant demographics is presented in Table 1. 

### 3.2. Definitions of Community Engagement 

There does not appear to be a standard definition of community engagement in the animal control and field services field, as definitions varied widely across the officers. Officers described several activities that they perceived to be community engagement, including but not limited to “telling their story on social media”; attending events; providing education for youth; doing presentations for other local organizations; driving around their community to build relationships with community members; and providing supplies or referrals to pet support services. 

***Community engagement is about being there every single day. It’s about being seen, being present, being known. It’s not an event, it’s a daily action (Participant 14).*** There are a variety of ways that the animal control and field services officers described or defined community engagement in their field. Some defined community engagement as, “making rapport” (Participant 28), “making a difference” (Participant 18), and “being personable” (Participant 24). Others defined it as, “being a positive resource to my community” (Participant 26) and “the ability to look past the traditional role of an animal control officer an enforcement official and more of as grassroots, let’s solve problems individual” (Participant 16). Moreover, some officers defined community engagement as a “two-way street” (Participant 10) with the community they serve. For example, one officer said community engagement included “the community being willing to help animal control or field service officers” (Participant 25).

Many officers explained that good and effective community engagement primarily consists of officers simply knowing the community they work in and asking for help from the community. Officers frequently noted the importance of informal relationship-building in community engagement and often described “being out in the community” and talking with the community. For example, one officer said, “community engagement to us is getting out into the community, talking to people, being proactive instead of just running around handing out citations and enforcing leash laws” (Participant 7). This theme is illustrated by a quote from another officer who elaborated on how their tactics for building relationships with members of their community (driving around the community, stopping to play basketball, and going to children’s lemonade stands) improved their community engagement efforts by humanizing them:

*I have seen that help tremendously just to see that there is a person behind this badge [...] I am no different than they are just because I put this [uniform] on. It’s a uniform, that’s all it is. I’m still a human. So, that has helped tremendously with my community. So, I’ve purposely gone to these areas that are normally considered dangerous for us and I’m trying to make the difference with them.*

(Participant 1)

Partnering with other local organizations and community members was described as another way to improve community engagement. Examples of these partnerships included working with law enforcement to train people on the link between harm to animals and harm to people, building relationships with key community members who “see everything” in the community, and participating in various task forces for their community. Officers reported partnering with local organizations and community members to distribute pet care supplies as an effective approach for increasing the recognition of animal control as a resource to help care for people and animals in crisis. Some of the officers interviewed for this study conveyed a need for better cooperation and communication across animal welfare and animal control. One officer suggested that organizations should institute memorandums of understanding between one another to increase cooperation and communication. They shared that “shelters not talking to each other is a huge detriment” (Participant 29) and the field should be “educated and open-minded to change” (Participant 9). Additionally, officers hoped for more partnerships with animal rescues and the removal of barriers to and requirements for the adoption of animals. Finally, officers described a desire for more uniformity in animal welfare and animal control to reduce confusion, misinformation, and variation in operations. They felt that the more they connect, network, and share with other organizations, the better the animal welfare field will be for animals and humans. 

### 3.3. Interpersonal Skills in Community Engagement 

Officers reported several interpersonal skills that impact community engagement in animal control and field services. They noted good/effective interpersonal skills to use in community engagement as “finding common ground”, “respect”, “active listening”, “patience”, “positivity”, and “compartmentalization.” Conversely, officers described bad or ineffective methods of community engagement as being “aggressive”, “abrupt”, or “confrontational.” 

***Talk to someone the way you would want to be talked to (Participant 10).*** The animal control and field services officers interviewed for this study explained that finding common ground is essential to engaging with the community they serve because the community is then “more likely to open up and talk to you in a positive way” (Participant 12). Moreover, the animal control and field services officers noted that respect was vital to community engagement. One officer shared the impact respect has on their relationship with the community:

*When I go on calls, the one thing that I try to keep in mind is a lot of times the person that I’m approaching has never been shown any respect or any courtesy by people, they’re looked down on by the general community. So, I try to always be very respectful. And most of the time when I leave cases, I’m a resource and they’ll call me if they see a stray dog or, you know, they are happy to see me.*

(Participant 23)

Additionally, officers explained that community engagement is doing everything possible for the people they serve. For example, one officer said, “don’t be a ‘no’ person when you’re out there” and noted that they work to help every person that comes to them with an issue, or they will refer them to someone who can provide the support needed (Participant 6). Moreover, officers shared that “being active listeners is the most important part of engaging with our community” (Participant 14). 

Officers noted that positivity and patience are critical to effective community engagement: “it seems like we get more cooperation when we start just being a lot more patient and just constantly kind of chipping away with positive advice.” (Participant 12). Further, officers shared that compartmentalization is an essential interpersonal strategy for being able to engage in effective community engagement:

*The other day I came off of two back-to-back injured [animal]s where, by the time I got back to the shelter, both of the dogs not only were injured, but it was neglect type injured. […] And in both of those incidences my staff did the best they could, but it was ultimately determined they needed to be euthanized. Well, for us as an officer, you’re walking out of that and onto your next call, trying to be positive. So, a lot of times when you’re hopping outta the truck, you’re trying to make sure that you are not carrying what just happened at the last couple of calls.*

(Participant 24)

Many officers highlighted how being aggressive could hinder community engagement. For example, one animal control officer said that “walking in and saying it’s my way or the highway; here’s your citation, and just walking away” really does not work in the community that they serve (Participant 22). Another officer explained:

*I think in this field it’s hard because everybody, every person is different, so you really have to cater to each person and their personality as you meet them. So, if you’re not doing that and you’re going up to them kind of being really abrupt and aggressive and confrontational it can be a little off-putting for people and that can definitely make them take a step back.*

(Participant 18)

### 3.4. Applications of Community Engagement 

There were a variety of animal protection issues and program areas where the animal control and field services officers described using “community engagement” strategies, including stray/lost and found/return to owner, community cats, wildlife, cruelty/neglect, dangerous dogs, events, social media, and pet support services (e.g., pet food pantry, veterinary services, dog houses, fences). 

#### 3.4.1. Stray, Lost & Found, and Return to Owner 

***When we return an animal that was running at large instead of taking it to the shelter, I think that’s building trust (Participant 9).*** Many officers described return-to-field (RTF) practices in the field as community engagement. Rather than impounding the animal, citing the owner, or charging fees, officers simply returned the animal to the owner while in the field. Officers expressed that trying to figure out where a lost dog belongs in a neighborhood before bringing it to the shelter, such as walking door to door or checking for a microchip, positively impacts trust with the community. Similarly, many officers shared that the community can help get lost pets home and believed this leads to effective community engagement. Officers explained that when community members find dogs, it is an opportunity for the animal control and field services officer to develop a relationship. Situations such as this allow officers to build upon existing community strengths. For example, one officer shared a story of leveraging existing strengths and relationships in the community they serve:

*Her dog got loose, but her neighbor, two houses, down came over, got her dog [and] was like, “Hey, I know you probably got the call, but I already took the dogs and put ‘em up. Look, I don’t want you to take the dogs, we looking out for each other.” So, that community togetherness, looking out for each other, even for their dogs is good.*

(Participant 28)

Adding or maintaining specific laws that would support a reduction in the number of animals who are stray, lost, or at large was a priority for officers. Some officers shared that they provide a grace period for people to obtain a license for their pet, which has proved effective in their community by increasing compliance with the requirement and preventing unnecessary fines and citations. Officers also proposed a mandatory spay/neuter ordinance in their community to reduce the number of animals at-large, the number of aggressive animals, shelter capacity issues, and overpopulation. Many officers believed that instituting a mandatory microchipping law or ordinance would help more than mandatory licensing in returning lost animals to their owners. 

Officers described certain laws as “unenforceable” and confusing to convey to the public. Such laws and ordinances often do not reflect what they believe is best practice in the field and officers advised that they be removed. Examples of these unenforceable laws included cat leash laws and nuisance dog barking ordinances. Officers also suggested removing exemptions for certain animals from the animal control laws and ordinances. For example, in one community that an officer serves, dogs used for hunting can run at large without receiving a citation, but if other dogs are found at large, their owner would receive a citation. Moreover, officers felt that animal control should eliminate unnecessary fees and fines to support keeping pets and people together. They shared that, very often, people cannot afford to pay these fees or fines and animal control needs more flexibility when they proceed with enforcement actions:

*I was required to charge people when I return their dog to them, even if it was licensed, even if it had a microchip. I was required to charge them, not with a citation, but a return fee of like $95 or something. And I was like, “Why?” Some people would be like, “I can’t pay it. Can you just take it.” And we’d have to take it.* *It’s little things like that I think are backward thinking.*

(Participant 6)

#### 3.4.2. Community Cats 

***There is literally zero reason if you see a healthy cat out on the street out in your neighborhood to scoop it up and bring it to the shelter. You’re probably stealing someone’s pet (Participant 7****).* Officers outlined how community engagement relates to community cats, free-roaming cats, and cat colonies. Many officers mentioned that their “trap neuter return” (TNR) programs were well received, good for the community, and positively impacted trust with the community. However, some officers described that the members of the communities they worked in do not support TNR programs and were upset that cats were returned near their properties. In addition, officers explained that their organizations’ “barn cat” programs positively impacted trust with the community by reducing the euthanasia of cats who may not be a good fit for placement in a family home. “Barn cat” programs are utilized by animal welfare organizations to rehome cats with families looking for a cat who can support them with tasks around their farm, such as rodent control [26,27]. Officers also noted mixed reactions to animal control policies discouraging officers from impounding or taking in stray, healthy cats. Some community members supported this policy, while others took some time to accept it and required explanations from animal control and field services. Another officer felt that the most important ordinance or law that needed to be removed in their community was picking up and impounding community cats. When discussing future directions for the field in community engagement, officers encouraged a broader implementation of TNR programs. Additionally, an officer shared that restrictions on how many community cats a caregiver can feed, where they can feed them, and when they can feed them have been effective in their community as they can support managing the line between a complete lack of regulation and the mass culling of community cats by animal control. 

#### 3.4.3. Wildlife 

***We always have a lot of requests for wild animals and that’s not something that we provide, but they feel like we should because their taxes pay for our employment (Participant 20).*** While the majority of the officers interviewed for this study focused primarily on their work with domestic companion animals, some officers touched on the impact of their agency’s wildlife management policies on community engagement. For example, officers shared that the people in their communities do not know or understand why animal control and field services did not address wildlife issues. As a result, animal control received pushback from the community when animal control would not or could not trap, move, or dispose of (euthanize/kill) wildlife. As a future direction for community engagement on wildlife issues, some officers described a need for agencies who are responsible for wildlife management to go to schools to deliver education and information on how to manage wildlife, while other officers expressed a wish for more general public education on wildlife. 

#### 3.4.4. Cruelty/Neglect 

***With real animal cruelty, like ill-treatment felony-type cases, we’re gonna get the search warrants, we’re gonna get ‘em. But when it’s a doghouse that just needs to be a better doghouse or vet care-that’s fixable (Participant 28).*** The animal control and field services officers shared that they utilize community engagement strategies when addressing cruelty and neglect issues. They explained that there are two sides to every situation and officers should be open-minded and “go in with fresh eyes” (Participant 15). The officers said that they often offered assistance and/or education to community members in these situations. Moreover, officers told the researchers that they offer the opportunity for the community members to comply with the law or ordinance before they proceeded to enforcement or punitive measures. Many officers noted that they treated “intentional” cruelty and neglect differently than “unintentional” cruelty and neglect. 

A small number of animal control and field services officers felt that all their current laws and ordinances were effective and did not need to be changed to improve community engagement. Some even stated that their community’s laws have served as examples for other communities. However, many officers described a desire to change their cruelty/neglect laws and wished the laws were stricter, clearer, and even potentially reclassified as felony offenses. One officer suggested implementing court-ordered psychological counseling for people convicted of animal crimes related to animal hoarding. Some officers believed the laws around adequate shelter and animal housing should be more strenuous. Other officers wished the definition of criminal neglect was clearer and more specific. Multiple officers explained that obtaining authorization for seizures of animals takes a very long time and, often, animals are left in unsafe conditions for longer than they perceive they should be. The sentiment among officers was that weak and ineffective laws prevent proper prosecution in court. 

Some officers felt that there needs to be more discretion in the animal control laws and ordinances to serve their community better. The need for discretion was described by one officer as those moments in the work when “You have your conscience of doing what’s right versus doing what the law tells you to do” (Participant 4). Some officers expressed that they needed discretion in their enforcement work mainly because their ordinances and laws are outdated and/or irrelevant. Another officer described what more discretion might look like in animal control and field services when they said, “I don’t think laws should be deciding outcomes. I think that the subject matter experts should be deciding outcomes. The laws can create the pathway and the punishment. But they shouldn’t be creating the outcomes for the animals” (Participant 14). 

Conversely, other officers believed that discretion should be removed from animal control laws and ordinances as it results in unequal enforcement and “picking and choosing battles.” For example, some officers felt that community members experiencing mental health challenges were being charged, convicted, and incarcerated for animal crimes, while others, whom the officers felt should go to jail, avoided penalty. Alternatively, other officers believed that animal control often seized animals without due process, and the community’s rights were violated. One officer advocated for greater checks and balances within animal control and field services to protect people’s rights.

#### 3.4.5. Dangerous Dogs 

***If somebody has a dog that’s aggressive and they can’t trust us to help make sure that they’re safe, then they’re not gonna have any trust in us (Participant 8).*** Officers also discussed utilizing community engagement strategies for dangerous dog issues. For the purpose of this study, the term “dangerous dogs” is used to encompass any dogs referenced by the officers as biting, dangerous, potentially dangerous, or vicious, as the requirements for a “dangerous dog” designation vary greatly across animal control agencies and jurisdictions. In addition to variations in the definition of “dangerous dogs” across agencies and jurisdictions, there are also notable variations in the outcomes for dogs deemed “dangerous” across the United States. Possible consequences for a “dangerous dog” depend on the laws and ordinances in a given jurisdiction and can include, but are not limited to euthanasia, mandatory relocation outside the jurisdiction, and mandatory containment and confinement. In general, the animal control and field services officers explained that they must “look at all sides” when dealing with dog bites and remain impartial and unbiased in these situations. 

The officers provided specific suggestions for how the laws and ordinances related to dangerous dogs could be improved. Many officers felt that dangerous dog ordinances should continue and, if anything, they could be more severe. However, some of the officers felt that the Breed Specific Legislation (BSL) in their community was unjust, a hindrance to their work, a bad reflection on animal control, a negative impact on community trust, and not beneficial. This theme could be illustrated by the following quote:

*Our dangerous dog [law] it’s overly punitive. It is discriminatory on income basis. There’s so many fees involved in it. It literally was set up so that when a dog is “bad”, we could just basically charge somebody out of ownership and I don’t like it and I try and get around it as much as I can, but because there’s so much citizen involvement in it, it can be very, very difficult.*

(Participant 14)

Many officers noted that the community gets upset with animal control and field services when they euthanize dangerous dogs. One officer wished that the euthanasia requirement would be removed from these laws to allow for more discretion from animal control and the court system. 

#### 3.4.6. Events 

***[Events are] really beneficial with trust because we go out there and they get to meet us and see who we are (Participant 18).*** A multitude of the officers interviewed by researchers for this study suggested that participating in and holding community events is good community engagement. These events included school educational events, city/county government-sponsored events, car shows, events with other city/county departments (fire department, police department, parks and recreation, etc.), silent auctions and fundraisers that benefit the animal welfare organization, and subsidized or free spay/neuter events, licensing events. Some officers even noted that participating in events is their definition of community engagement. A portion of the officers interviewed felt that attending events meant that animal control and field services officers are “present” in their communities, and some felt that attending events positively impacts trust in the community. Officers believed that community events allow them to engage with the public and that events personalize the officers to the public. Moreover, many officers expressed that any event that educates the public is good community engagement. In order to further improve community engagement, officers proposed the organization of town hall events, where both the community and animal control have the opportunity to speak and interact with one another. Officers also explained that adding adoption events and promotions throughout the year could help change public perception of animal adoption. They noted that their current “Foster to Adopt” programs should continue as they are positive for both the animals and the community.

#### 3.4.7. Social Media 

***You can only be in one spot physically in one moment, but you could be in a thousand [with social media] (Participant 29).*** During the interviews, animal control and field services officers often brought up social media/digital communications as good methods of community engagement. They noted that it provided a way to get information out to the public and can be particularly helpful in communicating what is going on in the shelter and in animal control (for example, posting a shelter capacity meter to tell the public how full the shelter is). Similarly, officers describe social media and other forms of digital communication as a way to return lost animals home to their families. 

A few officers noted that community members will advocate for animal control on social media. They described social media as a way to “tell the officers’ story.” Conversely, some officers felt that social media is not always helpful, and many community members complain about animal control and field services on social media platforms. In other words: “social media can be a blessing and a curse” (Participant 17). To engage well with the communities they serve, officers suggested that animal control and animal welfare organizations as a whole need to improve their use of technology and social media. 

#### 3.4.8. Pet Support Services 

##### Assistance, Education, and Information before Enforcement

***How do we not create a hardship for an owner? And how do we find a resolution that achieves the same outcome, but without any punitive aspect to it? (Participant 14).*** Almost all of the 29 officers interviewed told the researchers that they will offer the opportunity for community members to comply with laws and ordinances, provide them assistance, provide them information, or provide them education before they proceed to enforcement measures and that this is their definition of community engagement. This theme could be illustrated by one officer who summarized their approach as “educate first, comply second, and enforce third” (Participant 6). 

Most officers said that they will offer the opportunity for community members to comply (“I’ll do a follow-up”) with laws or ordinances by providing them assistance (“look at me as a person or an agency that would help”); information; or education (“public education first”) before they proceed to enforcement or punitive measures. For example, one officer shared that they consider community engagement to include sharing information that makes the laws, ordinances, and codes understandable for the community by putting them in contexts that are familiar to community members. Most officers interviewed for this study wanted more community education on existing resources, the difference between cruelty and neglect, pet ownership, laws and ordinances, and humane education for children. 

Furthermore, officers defined community engagement as providing assistance before proceeding to enforcement or punishment measures. Many officers shared the importance of connecting people to resources and that their definition of community engagement was helping both the animals and the humans in the household:

*I always like to say we wear multiple hats on the day. Just depends on who you’re talking to and getting them in contact with the right people, ‘cause, for example, you can go to a residence and start the conversation, “I’m there for the animal”. But usually, there’s something else going on. “Do you have enough food? Are there issues with your children? Have they gotten to a doctor lately?” Things of that effect. I mean, it’s staring at you right in the face. So, you really can’t deny that it’s there. So, if they’re not able to take care of themselves, they can’t take care of the animals. So, the way I look at it is being able to bridge that gap. If you can get them in contact with the services that they need, then a lot of those problems will fix themselves. I think there’s that preconceived notion that, “Oh, people are horrible. They’re doing bad.” Well, they actually love their animals. That’s one thing you talk to them they know everything about them. They just usually don’t have those connections to get to where they need to be. So, if you can help them, get across that bridge, then that’s really what that engagement’s about.*

(Participant 16)

##### Pet Food Pantries, Dog Houses, Fencing, and Veterinary Care

Officers described pet food pantries as a good community engagement strategy that helps achieve compliance with the law. In addition, pet food pantries were described as a good approach for involving community members in the work of their agencies by asking for food and supplies donations to distribute in the food pantry. Similarly, many of the officers interviewed for this study characterized providing free dog houses to people as a strategy to help build trust with the community. Officers shared that they will often provide a doghouse to a community member who does not have adequate shelter for their dog before utilizing enforcement methods. Furthermore, the animal control and field services officers felt that their organization’s fencing programs are an effective community engagement technique. Many of the officers expressed that they would prefer to help community members fix their fences before utilizing enforcement methods for correcting animal containment issues. Officers also described providing or connecting veterinary services as community engagement. Officers noted that free or low-cost licensing, microchipping, and/or veterinary care events build trust with the community. Some of the veterinary care services provided include low-cost or free spay/neuter, rabies virus vaccines, feline panleukopenia virus vaccines, and distemper virus and parvovirus vaccines. When the animal control organization could not provide these services, officers explained that they connected community members with veterinary services in their area and partnered with local organizations to provide these services to people externally. 

In order to sustain a focus on providing support in the community, there were several resources the officers felt needed to be added. Officers frequently cited improved shelter space and shelter resources as things to add, continue, or improve. Resources to add or continue also included equipment and supplies such as dog houses, fencing supplies, and pet food in officer trucks for assistance to the community. In addition, animal control and field services officers highlighted a need to add or continue providing community services for the people they serve. They believed there needs to be free or low-cost options for spay/neuter services and general veterinary care. Similarly, officers expressed a need to establish or continue pet food pantries for their community, create a monetary fund to use for community member access to pet care resources, provide emergency pet boarding and sheltering, and add resources for community cats. Officers noted a lack of accessible animal behavior and training resources in their communities and hoped to add those one day, citing behavior issues as a cause of animal surrender. Community support was touched on, with officers wishing their community would get more involved with each other and help one another with their animal-related issues. 

##### Organizational Philosophy, Policies, and Procedures

Another officer described how community engagement-focused policies and procedures are essential to doing the animal control work and “it would be a really hard job to do if you weren’t operating this way” (Participant 19). Another officer shared that when they do not support community members in their efforts to comply with the law before issuing punishments, it negatively impacts animal control officers’ relationship with the community:

*When I was in training with my [Field Training Officer], he was a little bit more on the stricter side. He didn’t let things slide, but for me, I really don’t believe in that. I believe in more of like if I have to do a follow-up in a couple of days, I’ll do a follow-up. And I’d rather do that than tell them, “All right. If you don’t change this by today, then we’re gonna have to write you a citation or a warning or something.” And I think that usually gets people irritated and it kind of gets them on the—I don’t wanna say bad side of us, but they don’t look at us the same way. They look at us as the people that are just here to write them warnings and citations trying to get on their bad side. So, I think that’s one of the biggest things that really doesn’t work.*

(Participant 4)

Organizational philosophy frequently came up as a factor that impacted an officer’s community engagement efforts. Officers felt that their organization’s emphasis on de-escalation techniques was effective and that alternatives to enforcement were their ideal future direction of the field. Others explained that the organizational philosophies of seeking to help somebody, keeping pets with their people, being open-minded, returning animals to owners in the field or diverting intake, and making sure they go out on every call should continue. Most officers described their desire for more organizations to adopt support over enforcement philosophies, while a few others advocated for the exact opposite. As a whole, they hoped that these philosophies “scale up” from organization-specific policies and practices to animal control-wide policies and practices. 

### 3.5. Community Engagement with Diverse Communities 

Officers from this study shared several community engagement strategies specific to working with individuals from diverse social and cultural backgrounds. Officers shared the importance of understanding the demographics of their community and how that may impact how people view their animals; respecting people’s religions; and providing resources and information in languages other than English. Many officers described an equal enforcement approach (“treat everyone the same” and “treat everyone equally”) and will enforce the same regardless of a person’s identity or background, while others described the need for an equitable approach to enforcement (“it’s really a socio-economic sort of situation”). 

***I feel like you have to be fair with the different demographics of people, but also then the different types of animals that you deal with (Participant 17).*** The animal control and field services officers discussed community engagement approaches they use specifically for engaging with populations that have been socially or economically marginalized in their community. Officers emphasized the importance of understanding the demographics of their community and how that may impact how people view their animals:

*It’s understanding the demographics [of your community]. For our department, we recently had a training on understanding your Hispanic Community. And not just understanding your Hispanic Community, but how they, as a community view their animals. And so, for myself, right behind me is a dog that’s asleep on the bed in the house. And you have other families that their dogs never come in the house, it’s a dirt yard the whole 10 yards. Does this mean I love this dog more than they love their dog? It’s a difference in viewing of how they view the dogs. The same thing comes down to say of Asian descent. Overseas, dogs are on the menu, so are cats. So, their viewing of the animals [might be] a little bit different, and you have to be careful when I go to approach them, I have to understand how they are viewing [animals]. And so that’s a big thing I think that’s been kinda lacking. When we got the training [on cultural sensitivity], I’m now able to go into those communities a little bit more and understand how they view the animals.”.*

(Participant 24)

Another officer highlighted the importance of respecting other people’s religious practices and working with community members to find animal control-related solutions that do not infringe on religious freedom:

*We have [community members] bring goats home and they’re going to slaughter them in their backyard and there is no law that says they can’t—that’s a religious practice or a cultural practice. You can do that [in the community I serve], but you can’t do it in plain view of all your neighbor’s kids. You always have to be mindful of people’s religions and their beliefs because I want mine respected. So, I wanna respect other people’s.*

(Participant 15)

Moreover, many officers said they “treat everyone the same” and “treat everyone equally” and enforce the same regardless of a person’s identity or background. One officer noted, “If you are not treating your dog well, I don’t care who you are” (Participant 21). Other officers explained that an equitable approach to enforcement is necessary-one that considers socioeconomic status, culture, ability, and access to resources:

*For instance, if we go back to the trailer park and maybe this person’s dog has gotten out three times and normally I’m like three times, “you’re gonna get a ticket”, but this person has no money. I know that they are not going to be able to pay a fine. I don’t think that that’s gonna be helpful to them. Let’s say maybe they need help figuring out a better fencing system, or, maybe they need help, I can put them in touch with a community group that could help them put up a fence or maybe some education on why it’s important to keep your animal on a leash or in your yard. I might go that route with that person versus writing the ticket. Whereas maybe if I’m up on the golf course, and this dog has been out three times and this person knows full well what the laws are, the person has a good fenced backyard, the person just doesn’t feel like they need to be putting their dog on a leash because they’re above the law, and then I might write a ticket to them. So I’ve treated two different parties in different ways, but I feel like I’ve still treated them fairly for their situation, right? They both got three chances. I’m gonna move forward with enforcement on this case because it’s really more a case of they just don’t care about the law, and they don’t want to follow it versus this where it’s really a socio-economic sort of situation with the other person.*

(Participant 17)

Some officers provided suggestions for how to improve their community engagement efforts with diverse communities. One officer suggested increasing the diversity of the animal control and field services department so that they can better serve their community. In addition, a few officers wished animal control would provide or would continue to provide information in languages other than English in order to improve communication. Others wished their organizations would institute rules and guidelines related to diversity, equity, and inclusion in order to improve trust with the community they serve. Finally, most officers emphasized the importance of making a shift in the profession to listening to the communities they serve:

*We’ve come to this place where we, for a very long time, felt like we were the end-all-be-all when it came to animals, we knew all the answers. And I think it’s really time for us to sit down and actually start listening to what our community needs.*

(Participant 14)

### 3.6. Challenges for Officers Participating in Community Engagement 

Officers described experiencing several challenges in their roles that can impact their ability to do effective community engagement. These included: verbal hostility; physical harm; mental health challenges (“your cup just starts running over”); and exposure to human and animal harm and neglect. Officers shared that they are underpaid, overworked, and their departments are understaffed (“no one wants to do the job”), underfunded, and under-resourced. Some noted a lack of support from their supervisors and leadership (“they should do more”); a lack of comprehensive, standardized training for their profession (“try to hold ourselves as an industry to a higher standard”); and challenges working with the animal welfare community (“just want to do it their way and no one else’s way”), other local organizations, government agencies, and elected officials (“wish they had a different perspective”). 

#### 3.6.1. Mental Health Support 

***We see just as much, and we deal with just as much as all law enforcement does (Participant 2).*** Throughout the interviews conducted with animal control and field services officers, participants detailed the challenges that they face in their role. Officers described witnessing acts of violence, harm, and neglect against animals and having to compartmentalize and remain calm and neutral. Some expressed how challenging it is to be unable to help the animals they feel they are there to protect. This included a shared sense of frustration with the legal barriers and investigative processes that elongate the time it takes to remove an animal from a situation they deem harmful. Others described the most negative part of their job as the euthanasia of animals. 

Consistently, officers touched on the mental health toll of their role and explained that it is a very tough job:

*You shouldn’t hate your job. You should go out there and wanna do your job and wanna make the difference that you started out wanting to do. And I can tell you that a majority of us don’t feel like that anymore because there’s no support from our higher-ups in the mental capacity. They expect us to be able to just block it, push it down and go on. And-after a while, it’s kinda like your cup just starts running over. You can’t take it anymore.*

(Participant 2)

Other officers told the researchers that they “take the job home with them” and described panic attacks and hallucinations as a result of their experiences in the field. Officers also expressed that they would become emotionally and mentally “numb” as more time passed on the job. Officers suggested that these mental health challenges, numbness, and burnout have direct negative impacts on community engagement. One officer explained, “you’re gonna hate your job. You’re gonna hate everybody you come in contact with and it’s gonna show and you’re gonna get in fights. You’re gonna have a lot of issues,” (Participant 21). 

The officers in this study shared an earnest desire for mental health resources for animal control and field services officers. They believed that increasing access to mental health resources for officers would help with community engagement because the officers sent into the field would be more “balanced.” They proposed a required yearly therapist visit for all staff as well as leadership check-ins with staff using a mental health checklist. In order to solve the perceived crisis in their profession, the officers said they need more support from their leadership. The officers also suggested the creation of an accessible phone hotline to call and receive mental health support specific to the field. Other officers explained that some law enforcement departments have peer-support programs to process and decompress from their experiences and they wished animal control and field services departments had something of a similar nature. 

#### 3.6.2. Staffing and Financial Resources 

Other challenges described by the officers included several staffing issues in their field. Officers shared that it takes “a special kind of person” (Participant 15) to be an animal control officer and “no one wants to do the job” (Participant 25). Another officer said, “a lot of people come into the business because they love animals, [and] you deal with animals, yes, but you have to know how to deal with people” (Participant 20). Specifically, officers believed that many of their staffing issues stemmed from being overworked and underpaid. Officers reported that departments are often understaffed which results in officers often feeling overwhelmed by the number of calls for service they receive and the need to triage calls, which in turn causes frustration in the community. Officers suggested that they often do not have time to attend or host community engagement events because they are so understaffed, under-resourced, and overworked. They also noted a lack of support from leadership at their organization and a lack of comprehensive, standardized training as significant challenges to their community engagement efforts. 

Officers also expressed challenges related to resources and often described their departments as underfunded and under-resourced. They expressed the desire to start new community programs and get equipment for officers but explained that resource barriers prevent the development of these programs and obtaining the equipment. Many officers also shared that they do not have enough shelter space to house all the animals that they must, and since seizures and confiscations of animals put an immense strain on resources, they try to avoid them when possible. However, some officers represented their agencies as possessing robust resources and mentioned that their community is very supportive with resources. 

Officers shared several suggestions for how to address the staffing and financial resource barriers to community engagement that they experience. Many officers suggested more rigorous qualification requirements for hiring animal control staff, including a standardized, formal education requirement to become an animal control and field services officer. They expressed a need for a psychological evaluation requirement and a more involved onboarding certification process. Similarly, many officers believe there is a need for more staff training, which includes hands-on training in the field, continued education, de-escalation classes, and training on the link between animal harm and human harm. An officer shared, “I would hope […] that we try to hold ourselves as an industry to a higher standard just like anyone else in public safety” (Participant 11). Some officers noted a degree of “red tape” in their organization that prevented them and fellow officers from receiving better training. In conjunction with a desire for increased training, officers expressed hope for a greater level of professionalism and standardization of the animal control and field services sector across agencies in the future. Officers proposed the creation of, and a requirement to be, “certified animal control officers,” receive standardized training no matter their agency, and/or require that all animal control officers be sworn officers in the eyes of the law. 

#### 3.6.3. Working with Other Agencies 

Furthermore, the animal control and field services officers interviewed for this study detailed various challenges with working with other agencies and organizations in their roles, such as animal rescues, animal shelters, domestic violence shelters, case managers, city councils, and other government agencies. Officers referenced difficulty working with other animal welfare organizations. Some officers reported that a local rescue group “steals dogs” from the public and others “just want to do it their way and no one else’s way” (Participant 11). One officer said that they get a good deal of “backlash” from other animal welfare organizations because they cannot save every animal, and they “do not take people’s animals away quickly enough” from the community’s perspective (Participant 12). Similarly, another officer described being criticized by the animal welfare community because they gave a community member a chance to get their dog vaccinated before issuing a ticket (Participant 28). Another officer said that some animal welfare organizations in their community complain that animal control does not have a longer stray hold for animals and that they do not pick up deceased animals. 

Moreover, some officers outlined the challenges they face when working with government agencies, government departments, and elected officials. They often felt that these government entities and officials do not support them adequately, are unhelpful, and could do more. One officer explained the challenges they face with their local city council:

*I think we have to work with city council. I wish they had a different perspective of what we do. I think they think we just go out write tickets and put bad people in jail where we are truly out there for the community. And I wish our city council members would understand that they’re supposed to be there for their community and what they’re doing is stifling their community and the pets rather than wanting to improve their city. I just wish the city council here would be more open to the public rather than thinking that they’re protecting the public with all the things that they’re doing in the animal field.*

(Participant 13)

Other officers echoed similar sentiments that their community is very aware of city government decisions, and they need to have their voices heard. They noted that if the community is not listened to and “shut down” by government agencies and/or elected officials, then animal control receives immense pushback (Participant 22). Conversely, a few officers felt that government agencies, departments, and elected officials in their community do support them and can be helpful in gaining financial resources and supporting legislation that animal control deems necessary.

#### 3.6.4. Misperception of the Profession 

Officers felt that their community has a negative perception of animal control and field services officers, which is often a barrier to community engagement. They explained that the community often views them as “dog catchers”, and thinks they are going to “snatch people’s pets” and “kill everything”. However, many officers expressed that effective community engagement helps to improve the public’s perception of animal control and field services and that an assistance before enforcement approach to animal control in their community has improved the perception of animal control and field services. 

***They just see us as dog catchers (Participant 21).*** With immense frequency, officers detailed the impact of the community’s perception of animal control and animal control officers on community engagement. Overwhelmingly, officers felt that their community had a negative perception of animal control and animal control officers. Generally, officers noted a lack of trust in them from their community. Many officers believed that their community did not have a good understanding of the role of animal control and field services officers and sometimes did not even know that they existed. A consistent theme that arose from officer narratives was a refutation of the idea that they are “dog catchers”, “animal stealers”, and an “animal dumping ground”: “A lot of people don’t take Animal Control agencies seriously. They just see us as dog catchers. That’s a big stigma that’s hard to get rid of. That hurts us immensely with community engagement” (Participant 21). Similarly, about one-third of the officers also said that the community thinks they are going to “snatch people’s pets” and “kill everything”. Many presumed that this perception stems from the problematic historical practices of animal control. One officer elaborated on their method for countering this perception of animal control:

*I try to build trust by going to schools, speaking to the kids, trying to educate what we’re here for, why we do this job, why I got in this field. And the animal control from back in the past—I try to paint it as a better future. We’re not just here to euthanize your animals. We’re not just here to cause problems. We’re here to fix the problem. It’s what we’re here for. And I think we have really, really emphasized that here. And it’s really turning people’s heads and making them realize that we’re not the bad guys. We’re here to help.*

(Participant 3)

***You are their voice (Participant 2****).* A majority of the officers interviewed for this study defined their role in the community as public servants. One officer explained the evolution of his understanding of this role and his identity as an animal control officer over time:

*Does the badge make the person, or does the person make the badge type thing? And I think it’s the person who makes the badge, realizing that you know, when you’re new to the field, I think you just come in with your guns blazing, I’m gonna fix it through tickets and seizures and all that stuff. And then you realize that’s not how it works. And so, I think it’s just a matter of getting that experience to understand how communities function as a whole, and then what’s your role in that to make things better for everybody that’s involved.*

(Participant 16)

Other officers felt that their role is to enforce laws with discretion and to be a community representative in difficult situations so that the community doesn’t have to be. For example, in neighbor disputes when people are worried about sharing information about an incident, one officer always explains that it’s them who will “take the heat in that situation” (Participant 27). Similarly, officers identified their role in the community as a neutral party that helps resolve conflicts without judgment. Moreover, officers felt that their role is to protect the animals and be their voice (“voice for the voiceless”). Participant 2 shared, “you are their voice, you are taking a stand for them in the legal aspects and saying, ‘Hey, you know, yeah, these are ‘property’, but they also are animals. They are living, breathing things that need justice too’”. Other officers believed their role is to “protect” and “preserve” public safety and sometimes this means making difficult decisions for animals and humans alike. In addition, officers believed that their role in the community is to have a balance of enforcement and assistance. Notably, a few officers perceived their role to be keeping people and their pets together and connecting them to resources. Relatedly, educating the public was often described as a role that the officers hold. 

***People right off the bat think we’re cops (Participant 21).*** Most of the officers interviewed for this study said that the community does not trust them because they look like they are, or they actually are, law enforcement officers. Officers shared that, from their perspective, looking like law enforcement officers, or being law enforcement officers, hurts community engagement in animal control:

*I think that animal control is one of the jobs in the world where you have to be able to wear many different hats. And if the hat that everybody sees is just as an officer, it makes it really hard to get to the places within the community that you really need to help people.*

(Participant 14)

However, some officers described benefits associated with operating within a law enforcement agency, including access to equipment (bulletproof vests, firearms, etc.), training, and being “taken more seriously”. Many officers also expressed the potential benefits of looking to law enforcement agencies that have been using “community policing” models to inform their community engagement strategies. One officer explained:

*There’s a lot more emphasis on training and community involvement. Community policing has been a big one in law enforcement and I think it’s starting to work its way over to animal control and that’s what it needs to do.*

(Participant 11)

Some officers expressed a desire to be allowed to carry firearms to protect themselves in the field. Much like the “red tape” described in accessing more training, officers identified bureaucratic barriers to obtaining authorization to carry a firearm or other weapons, such as a baton or pepper spray. Conversely, one officer shared that they do not believe animal control and field services officers should carry firearms or even be a part of law enforcement. 

This potential overlap between animal control and law enforcement is further complicated by the perception that law enforcement often looks down on animal control and field services officers (“the redheaded stepchild”). Officers in this study described how they wished that law enforcement would view them as important contributors to public safety. 

***Seeing officers repairing fences, helping get pet care, dropping off dog food to owners who need it—I think that really has been changing the outlook (Participant 14).*** Some officers in the study felt that they witnessed or are witnessing the evolution of the field and a positive change in the perception of animal control and field services. Officers described that the community that they serve increasingly respects them and views them as “helpers”. Moreover, officers expressed a belief that community engagement and an assistance before enforcement approach to animal control in their community has improved the perception of animal control and field services. Some noted that this stems from convincing the community that they are “not just here to take their dogs”. One officer detailed the shift in outlook from the community they serve:

*I think more and more so our community is starting to realize that we’re not out there just trying to take away their dogs. I mean, in reality, we don’t have anywhere to put their dogs, even if we did, like it’s not something we want to be doing. And so, seeing officers repairing fences, seeing officers helping get pet care, seeing officers drop off dog food to owners who need it. I think that really has been changing the outlook.*

(Participant 14)

Officers believe that community engagement efforts have improved the public’s perceptions of animal control and field services: “They see us, they don’t flip us off anymore. When I first started there, that’s kind of the attitude citizens had. Now, it’s like, ‘oh no, leave him alone. He’s here to help’” (Participant 21). 

### 3.7. COVID-19’s Impact on Community Engagement 

The COVID-19 pandemic that began in early 2020 had an overwhelmingly negative impact on community engagement according to the officers. Officers said programs, clinics, and all other public-facing community events had to stop. As a result, it was difficult for the public to access pet services appointments in the community; and staffing and volunteer numbers at animal control and field services agencies decreased. However, some officers said that the COVID-19 pandemic had a few positive impacts on community engagement. 

***They were going without, for themselves, for their animals (Participant 1).*** Given that the interviews for this study were conducted in the first half of 2022, many officers brought up the coronavirus (COVID-19) pandemic and its impact on community engagement in their field. Their perception of the pandemic’s impact on community engagement was overwhelmingly negative in nature, with just a few positive impacts shared with the researchers. 

Officers explained that their behavior (training) programs, vaccine clinics, and all other public-facing community events had to stop due to the pandemic. In addition, they noted that the pandemic had made it very difficult for the public to access any veterinary, spay/neuter, and grooming appointments in the community, and the virus has decreased staffing and volunteer numbers, further limiting the services offered by animal control. This presented a significant problem when coupled with the fact that the officers’ communities experienced exacerbated social, economic, and healthcare challenges due to the coronavirus pandemic. One officer detailed the nature of the issue:

*They were taking great care of their animals, but it was getting to a point where I had a pretty good amount of people that were going without, for themselves, for their animals. So, I worked it out with them and actually started bringing food. I bring dog food, cat food, litter, and just tell them, you know, “Hey, here’s my cell phone number, if you need anything, call me.” And then I got in with our local missions and local churches to help provide human food and clothing as well for these situations.*

(Participant 1)

Officers shared additional challenges that occurred during the pandemic, including receiving more barking dog complaints from people working from home and officer training being converted to an online format, eliminating hands-on training in the community. The pandemic also significantly delayed cruelty and neglect cases progressing through the legal system. 

Conversely, a few officers noted positive impacts of the pandemic on community engagement. They said that it helped them focus on social media messaging and transparency within the department as social media was vital for public engagement during the pandemic. One officer shared: “I mean, social media is the biggest point where I see all the engagement happening. Especially during COVID, that’s how they were all talking to each other and that’s how they would put in calls and requests for service” (Participant 21). One officer described how the pandemic helped their department to a small degree because more people were working from home, leading to larger volunteer potential from the community. 

## 4. Discussion

The goal of this study was to summarize animal control and field services officers’ perspectives on community engagement. The definition of “community engagement” varied widely across the twenty-nine participants. There are a variety of animal protection issues for which animal control and field services officers are using “community engagement” strategies, including cruelty and neglect, dangerous dogs, stray/lost and found/return to owner, community cats, and wildlife. While it is encouraging to see how animal control and field services agencies are attempting to integrate community engagement concepts into their work, the substantial variation in strategies described as community engagement represents a significant barrier to future evaluation of the effectiveness of this approach to preventing and responding to animal cruelty and neglect. These findings highlight a critical need for research to evaluate best practices and establish evidence-based approaches for more effective community engagement. 

### 4.1. Practice Implications 

The growing diversity of the U.S. population paired with historic and ongoing social and economic marginalization increasingly calls for nuanced approaches to public safety and well-being [10,28]. Community engagement is uniquely positioned to achieve this nuance in animal protection, given that it supports organizations and policymakers in identifying community-specific needs and proposed solutions. Currently, there are programs in the United States that are utilizing community engagement strategies to improve the health and well-being of animals and humans alike. Some of these programs include the Humane Society of the United States’ Pets For Life program (PFL), Companions and Animals for Reform and Equity (CARE), Best Friends Animal Society Community-Supported Sheltering Initiative, and American Pets Alive’s Human Animal Support Services program (HASS) [10,29,30,31,32]. More research is needed to assess current or future approaches to community engagement when they are implemented in animal control and field services.

Animal control and field services professionals who participated in this study often referred to human policing as a field they model for practices to inform their community engagement efforts. Integrating community engagement strategies in the human public safety field has largely involved making a shift to prevention-focused policing, including implementing a “community policing” model [33]. The field generally describes community policing as an approach that allows public safety professionals to engage in productive dialogue, form strong partnerships, and identify meaningful solutions, such as connecting individuals in the communities they serve with the resources and support they need, which can all be critical components of crime prevention and community safety [34,35]. This proactive engagement is particularly important when attempting to connect with populations who have been socially and economically marginalized that tend to distrust the police due to historic and present-day issues of systemic discrimination [34,36,37,38,39,40,41,42]. 

The growing body of research on the effectiveness of community policing has identified mixed results [43,44]. Crowl (2017**) [45]** found that the research to date has focused on several outcome measures that have significant overlap with some of the topics identified by the animal control and field services officers in this study. The impacts of community policing on officer job satisfaction, fear of crime among citizens, and crime reduction are still unclear [45]. However, the research indicates that community policing has consistently improved citizen attitudes toward the police and improved officers’ perception as a legitimate authority [46,47]. This is important because residents’ perception of officers can improve the community’s trust and satisfaction with the police, which can then improve quality of life and overall fear of crime [48,49,50,51]. 

Despite the potential positive impacts of community policing models, it is important to acknowledge the harm that has been perpetuated through community policing or other “prevention-focused” policing models. Several issues have emerged since the adoption of community policing models, including dramatic increases in racial bias and police violence [52,53]. Earlier iterations of the community policing model included “broken windows” policing, “quality-of-life” policing, and “order-maintenance” policing [52,54]. Each of these models has traditionally relied on the use of “high frequency” and “discretionary” policing to prevent the spread of crime, which have all been well-documented for their disproportionate impacts on populations that have been socially and economically marginalized [55,56,57,58,59]. To date, enforcement of animal cruelty and protection laws has perpetuated many of these same issues of racial bias and disproportionate impacts on individuals who have been socially and economically marginalized in the United States [12,60,61]. Community policing models have routinely failed to acknowledge how historical, geographic, social, and cultural differences have shaped community-police relations [2,41,62]. They have also failed to address the systemic causes of crime, poverty, and mobility restrictions, including racial discrimination and disparities in generational wealth accumulation [52,63]. Further, the implementation of community policing has potentially perpetuated economic marginalization by increasing rates of fines and fees [56]. Several studies have demonstrated how animal cruelty enforcement has occurred in communities with higher rates of economic stress, vacancy, and other crime [8,64]. To this end, efforts to integrate community engagement strategies in animal control and field services should proceed with recognition of this historical context and dedicate intentional action towards identifying how to ensure similar harm is not perpetuated by animal control and field services agencies. 

There are several other approaches to providing support to communities that do not involve enforcement agencies. For example, many enforcement agencies have moved towards dispatching social workers, mental health care providers, or survivor advocates to calls involving individuals in crisis, rather than law enforcement officers [36]. More generally, the literature indicates that prevention and support-focused programs (e.g., harm reduction for substance use disorders, restorative justice in education settings) are more effective and sustainable than punitive approaches (e.g., zero-tolerance policies, “three strikes” rules, mandatory minimum sentencing) [65,66]. Almost all officers interviewed in this study shared that they prefer to offer the opportunity for community members to comply with any relevant laws or ordinances, by providing community members with assistance, information, or education before they proceed to enforcement as a component of their community engagement efforts. Coulter et al. (2022) similarly found that implementing problem-solving strategies as an alternative to traditional criminal-justice tools may be both more effective and ethical when working with people with socially and economically marginalized identities. Furthermore, literature on the spectrum of animal harm severity suggests that criminal-justice responses are not uniformly appropriate for every neglect and cruelty situation [4]. For example, a person who is having difficulty caring for their animals due to a mental health condition would likely benefit from social services and problem-solving, rather than carceral punishment [4]. These support-focused efforts may be in tension with others in the animal welfare field who are advocating for increased criminal sanctions and reporting for animal cruelty and neglect, which are largely justified based on the presumed “link” between violence toward animals and violence toward humans [4,10,60,67]; A rigorous evaluation of which strategies are more effective in animal protection efforts is needed.

### 4.2. Barriers to Community Engagement in Animal Control and Field Services 

The animal control and field services officers in this study described many barriers to implementing and sustaining community engagement programs. These barriers included: obtaining and maintaining support from leadership; funding or staffing limitations; challenges related to developing and maintaining partnerships with other community-based organizations; the need to add, change, or remove laws or organizational policies; stigma or misconceptions about the role of animal control and field services in their community (e.g., the “dogcatcher,” being seen as police/law enforcement); and the need for training and mental health support for officers. These barriers are consistent with other studies in Canada that identified barriers to effective animal protection work. These barriers included inadequate resources, vicarious trauma from exposure to human and animal suffering, communication and information gaps, a culture of normalized disrespect towards officers, and an increased risk of gender-based discrimination [68,69].

The scientific literature indicates that an effective shift to prevention-focused policing requires a true organizational transformation in the values, mission, training, policies, and performance evaluations of the enforcement agency, and not just the basic procedures for service delivery [70]. Therefore, the barriers to obtaining and maintaining support from leadership, developing and maintaining partnerships, needing sufficient funding and staffing, and resistance to policy reform at many levels, all represent significant sustainability issues that the animal control and field services field will need to take steps to address when implementing community engagement strategies. Tangible steps to address these barriers might look like diversifying the animal control and field services profession and reallocating financial and staff resources in municipalities [10,71,72]. 

The need for training and mental health support for officers cannot be overlooked when integrating community engagement into animal control and field services. A recent study conducted by Stevenson & Morales (2022) [73] found that Canadian animal protection and welfare workers felt unprepared and overextended in their work from a mental health standpoint, which demonstrates a critical need for a more supportive agency atmosphere and the implementation of trauma-informed policies and practices. The need for improved support for officers has also been prevalent in the discussion of sustaining effective community policing [74,75,76,77].

Finally, there is a critical need for transparency and oversight of data collection on the frequency and outcomes of animal control and field services activities. Jenkins and Rudd (2022) [78] further highlight the need to collect data that provides the ability to conduct detailed analysis by intersectional identities (e.g., race/ethnicity, disability, gender, and sexual orientation). By making records public and requiring agencies to collect more complete data in all their activities, including their community engagement efforts, policymakers and enforcement agencies can monitor and address any patterns of misconduct in animal control and field services [52]. This data collection could also be a vital tool for evaluating the effectiveness of current or future community engagement approaches when they are implemented in animal control and field services.

### 4.3. Limitations

While the current study has contributed to a better understanding and captured a wide range of views and experiences of community engagement among animal control and field services officers, it has several limitations. The sample size for this study was relatively small (29 officers) and should be taken into account when considering the representativeness of this study for the entire animal control and field services profession. Further, these interviews were conducted with members of the National Animal Care & Control Association (NACA), the national professional membership organization with a focus on education and training, and there may be differences in the perspectives and lived experiences of individuals who choose to be members of such an organization from those who are not members.

## 5. Conclusions

This study fills a critical gap in the research by identifying animal control and field services officers’ perspectives on how to integrate community engagement strategies in their animal protection efforts. Future research and policymaking should seek to establish a consistent definition of community engagement in animal control and field services that can then be optimized for specific communities through rigorous evaluation. 

## Figures and Tables

**Table 1:** Demographics of the Sample (*n* = 29).

Participant Demographics		Frequency	Percentage
**Gender**	Female	16	55%
	Male	12	41%
	Transgender	1	3%
**Race**	Asian	2	7%
	Black	1	3%
	Hispanic/Latino/a/e	2	7%
	White	23	79%
	Two or More Races	1	3%
**Organization Type**	Municipal Agency	22	76%
	Non-profit Agency with a Government Contract	6	21%
	Other	1	3%

## Data Availability

Not applicable.

## Appendix A

**Interview Guide**

1. Why did you choose a job in animal control and field services?
2. What strengths does the community you serve possess for supporting people and animals?
3. What challenges does the community you serve experience in supporting people and animals?
4. How do you define “community engagement” as an animal control and field services officer?
5. Please describe your current approaches to engaging with community members in your work in animal control and field services. Please use specific examples of strategies you have found that work well for your community and strategies that do not work in your community.
6. How do you build trust with the community that you serve?
7. Do you feel that any of your agency’s current policies/programs/practices positively impact the trust between you and the community, in any way? If yes, please provide specific examples.
8. Do you feel that any of your agency’s current policies/programs/practices negatively impact the trust between you and the community, in any way? If yes, please provide specific examples.
9. What does being fair and impartial in animal control/field services look like to you? Please provide a few specific examples.
10. What, if any, animal control policies/ programs/ requirements or resources need to be continued to better support people and animals in the community you serve? Do any need to be added?
11. What, if any, animal control policies / programs/ requirements or resources need to be removed to better support people and animals in the community you serve?
12. The purpose of this study is to understand perspectives on the strengths and challenges of current approaches in animal control and field services to engaging with community members. What did we not ask about that you think is important to share?
